# Effect of DAPAgliflozin on Myocardial Fibrosis and Ventricular Function in Patients with ST-Segment Elevation Myocardial Infarction—DAPA-STEMI Trial

**DOI:** 10.3390/jcdd12060220

**Published:** 2025-06-11

**Authors:** Luis Ortega-Paz, Claudio Laudani, Alessandro Sionis, Pablo Vidal-Cales, Victor Arevalos, Rut Andrea, Carlos Igor Morr, Oriol De Diego, Emilio Ortega, Francisco-Rafael Jimenez-Trinidad, Ana Paula Dantas, Dominick J. Angiolillo, Manel Sabaté, Jose T. Ortiz-Pérez, Salvatore Brugaletta

**Affiliations:** 1 Division of Cardiology, College of Medicine, University of Florida, Jacksonville, FL 32206, USA; claudani313@gmail.com (C.L.); dominick.angiolillo@jax.ufl.edu (D.J.A.); 2 Division of Cardiology, Azienda Ospedaliero Universitaria Policlinico “G. Rodolico-San Marco”, University of Catania, 95123 Catania, Italy; 3 Cardiological Intensive Care Unit, Cardiology Department, Hospital de la Santa Creu i Sant Pau, Sant Pau Biomedical Research Institute (IIB Sant Pau), 08041 Barcelona, Spain; asionis@santpau.cat; 4 Department of Medicine, Autonomous University of Barcelona, 08193 Barcelona, Spain; 5 Division of Cardiology, Centro de Investigación en Red de Enfermedades Cardiovasculares (CIBERCV), 28028 Madrid, Spain; 6 Cardiology Department, Cardiovascular Clinic Institute, Hospital Clínic de Barcelona, University of Barcelona, 08193 Barcelona, Spain; pvidalcales@gmail.com (P.V.-C.); varevalos88@gmail.com (V.A.); randrea@clinic.cat (R.A.); morr@clinic.cat (C.I.M.); dediego@clinic.cat (O.D.D.); eortega1@clinic.cat (E.O.); masabate@clinic.cat (M.S.); jtortiz@clinic.cat (J.T.O.-P.); sabrugaletta@gmail.com (S.B.); 7 División de Medicina Cardiovascular, Hospital de Clinicas, Universidad Nacional de Asunción, Asunción 2169, Paraguay; 8 Department of Biomedical Sciences, Institut de Investigacions Biomèdiques August Pi i Sunyer (IDIBAPS), 08036 Barcelona, Spainadantas@recerca.clinic.cat (A.P.D.); 9 Department of Biomedical Sciences, School of Medicine and Health Sciences, University of Barcelona, 08193 Barcelona, Spain; 10 CIBER de Fisiopatología de la Obesidad y Nutrición (CIBEROBN), Instituto de Salud Carlos III, 28028 Madrid, Spain

**Keywords:** dapagliflozin, myocardial fibrosis, ventricular function, left, myocardial infarction, sodium–glucose transporter 2 inhibitors, cardiac magnetic resonance, randomized controlled trial, ventricular remodeling, heart failure, extracellular space

## Abstract

**Background:** Myocardial fibrosis leads to ventricular dysfunction and worsened prognosis, especially after ST-segment elevation myocardial infarction (STEMI). Sodium–glucose cotransporter 2 inhibitors (SGLT2is) offer cardiovascular benefits by reducing markers of myocardial fibrosis and fibroblast activity. However, the effects of SGLT2i on myocardial fibrosis deposition among STEMI patients undergoing primary percutaneous coronary intervention (PCI) have not yet been evaluated. **Study and Design**: The effect of DAPAgliflozin on myocardial fibrosis and ventricular function in patients with STEMI (*DAPA-STEMI*) trial is a phase III, multicenter, randomized, double-blind, placebo-controlled trial. The study aims to assess the effects of dapagliflozin on myocardial fibrosis and ventricular function, evaluated using cardiac magnetic resonance (CMR), in STEMI patients undergoing primary PCI. Eligible patients were 30 to 85 years old and exhibited a left ventricular ejection fraction ≤ 50%. A total of 120 patients with STEMI were expected to be randomized 1:1 to receive dapagliflozin 10 mg or placebo daily for six months. The primary endpoint is the change in the extracellular volume fraction of the remote myocardium from baseline to six months, as measured by CMR. The secondary endpoints include changes in the circulating C-terminal propeptide of type I procollagen, N-terminal propeptide of type III procollagen, and Galectin-3 from baseline to six months. The study was stopped prematurely due to slow recruitment, with 54 enrolled patients, limiting the statistical power to detect changes in the primary endpoint between groups. **Conclusions:** The DAPA-STEMI trial will provide insights into the impact of dapagliflozin on myocardial fibrosis and ventricular remodeling in patients with STEMI undergoing primary PCI. **Clinical Trial Registration Unique Identifier**: NCT06619600

## 1. Introduction

Myocardial fibrosis is the expansion of the cardiac interstitium by due to the deposition of extracellular matrix proteins. There are two main types of myocardial fibrosis: diffuse interstitial fibrosis and focal replacement fibrosis [1]. Diffuse interstitial fibrosis is the widespread deposition of collagen fibers between myocytes, usually seen in response to chronic conditions such as hypertension or heart failure [1]. On the other hand, focal replacement fibrosis is the deposition of scar tissue that replaces dead or damaged myocytes, often following events like myocardial infarction (MI) [1]. Of note, both diffuse and focal fibrosis contribute to ventricular dysfunction and worsen long-term prognosis [1,2]. Patients with ST-segment elevation MI (STEMI) experience both focal replacement fibrosis in the region of the MI and diffuse interstitial fibrosis in the remote myocardium as interstitial fibrosis is triggered by ischemia and subsequent inflammation [3].

Sodium–glucose cotransporter 2 inhibitors (SGLT2is) provide cardiovascular benefits beyond glucose control, reducing heart failure hospitalizations and cardiovascular mortality in patients with or without type 2 diabetes mellitus (T2DM). Preclinical studies indicate that SGLT2is modulates myocardial fibroblast activity, decreasing fibroblast migration and inhibiting profibrotic pathways [4]. Additionally, in individuals with T2DM and stable coronary artery disease (CAD), SGLT2is have been associated with a reduction in myocardial extracellular volume (ECV), a marker of myocardial fibrosis, as measured by cardiac magnetic resonance (CMR) [5]. However, the effects of SGLT2is in remote myocardial fibrosis and ventricular function in patients with STEMI undergoing primary percutaneous coronary intervention (PCI) have not been studied.

The effect of DAPAgliflozin on myocardial fibrosis and ventricular function in patients with STEMI (DAPA-STEMI) trial aims to assess the effect of dapagliflozin on myocardial fibrosis and ventricular function in patients presenting with their first clinically diagnosed STEMI, compared to placebo, by means of CMR imaging and circulating biomarker assessment.

## 2. Methods

### 2.1. Trial Organization and Sources of Funding

The DAPA-STEMI trial was conceived and designed by the Trial Steering Committee (Supplementary Materials). It is sponsored by the Spanish Society of Cardiology and registered in the European Union Drug Regulating Authorities Clinical Trials Database (EudraCT, 2018-003105-25) and ClinicalTrials.gov (NCT06619600). The lead site is the University Hospital Clinic of Barcelona. Funding for the trial as well as dapagliflozin and the matching placebo is provided by AstraZeneca (ESR-19-14489). The sponsor and funder had no role in the design or conduct of the trial and will not be involved in data analysis or interpretation. The trial protocol and any substantial amendments were approved by the Spanish Agency of Medicines and Medical Devices and the Research Ethics Committee of the University Hospital Clinic of Barcelona (HCB/2018/0960). The current protocol version is 2.1, dated 21 September 2021. Although the study planned to enroll a total of 120 patients, enrollment was terminated early due to slow recruitment, resulting in a final sample size of 54 patients. The study was conducted in accordance with the Declaration of Helsinki. Written informed consent was obtained from all participants prior to any study-related procedures. A Data Safety Monitoring Board (DSMB) oversaw participant safety throughout the trial (Supplementary Materials).

### 2.2. Protocol Amendment

Two protocol amendments have been made. The first amendment was made on 25 September 2020, which allowed the inclusion of participants with and without T2DM. This amendment was introduced to align with the results of the Study to Evaluate the Effect of Dapagliflozin on the Incidence of Worsening Heart Failure or Cardiovascular Death in Patients With Chronic Heart Failure (DAPA-HF) trial, in which dapagliflozin was shown to reduce cardiovascular events (including worsening heart failure and cardiovascular death) in both patients with and without diabetes [6]. Notably, this amendment was implemented prior to the start of patient screening and enrollment.

The second amendment, dated 18 June 2021, was made to harmonize the selection criteria with the Dapagliflozin Effects on Cardiometabolic Outcomes in Patients with an Acute Heart Attack (DAPA-MI) trial by adjusting the screening criteria to include patients with a left ventricular ejection fraction (LVEF) of ≤50% [7]. Additionally, the window for performing baseline CMR was extended to up to 15 days after the index event. This amendment was implemented after the enrollment of only three patients.

### 2.3. Patient Eligibility

The participants in the study had to meet all of the inclusion criteria. Those who met any of the exclusion criteria were not enrolled in the study.

#### 2.3.1. Inclusion Criteria

1. Patients between 30 and 85 years of age.
2. Patients with a first myocardial infarction with ST-segment elevation, documented in an ambulance or a cardiac catheterization laboratory (ST-segment elevation ≥2 mm in at least two contiguous leads) <12 h after the onset of symptoms that last ≥20 min, undergoing primary percutaneous cardiac intervention. The target lesion must be a de novo lesion located in a native vessel.
3. The patient understands and accepts clinical monitoring and CMR.
4. The patient must be hemodynamically stable (Killip classification 1) at the time of the initial CMR.
5. A left ventricular ejection fraction ≤50% at baseline echocardiogram.

#### 2.3.2. Exclusion Criteria

1. Pregnancy or lactation in women.
2. Type 1 diabetes mellitus.
3. Previous treatment with SGLT2is.
4. Severe liver disease (Child-Pugh C).
5. Kidney disease defined as stage III or worse (eGFR < 45 mL/min).
6. Systolic blood pressure <90 mmHg at the screening visit.
7. Malignancy (receiving active treatment) or other life-threatening diseases.
8. Any contraindication to CMR (e.g., claustrophobia; cerebrovascular implants; metal implants; penetrating eye injury; exposure to metal fragments in the eye that require medical attention; or hemodynamic or electrical instability).
9. Previous complicated urinary tract infection in men or repeated urinary infection in women.
10. Treatment with fibrinolytic therapy.

### 2.4. Screening

Transthoracic echocardiograms were performed during routine clinical care for participants presenting with their first clinically diagnosed STEMI undergoing primary PCI to identify those with an LVEF ≤ 50%, measured by Simpson’s Biplane or estimated by visual assessment. Participants meeting the inclusion criteria and none of the exclusion criteria were approached by study investigators at each site to obtain consent. Consenting participants underwent a baseline CMR scan. Participants with an LVEF ≤ 50% on CMR were randomized, while those with an LVEF > 50% were excluded. To minimize the influence of baseline myocardial fibrosis developed before randomization, being maximal between 2 and 8 weeks after the index event [8], the baseline CMR was required to be performed within 15 days following STEMI, preferably before hospital discharge.

### 2.5. Trial Procedures

#### 2.5.1. Randomization and Blinding

Participants were randomized in a 1:1 ratio to receive dapagliflozin 10 mg daily (intervention) or a placebo (control). The randomization schedule was generated by an independent statistician using a computer-based program for stratified randomization, incorporating two stratification variables (i.e., presence of T2DM and infarction site) to ensure a balanced distribution across treatment groups. Participants were enrolled by the study investigators at each site after eligibility was confirmed and informed consent obtained. The trial pharmacist, who had exclusive access to the allocation codes, assigned participants to the intervention or control group. Randomization was stratified by the presence of T2DM (defined as an established diagnosis, glycated hemoglobin [HbA1c] ≥ 6.5% at index admission, fasting blood sugar ≥ 126 mg/dL, or 2 h blood sugar ≥ 200 mg/dL) and localization of the MI (anterior vs. non-anterior) [9]. Before randomization, baseline assessments were conducted, including weight and height measurement, a physical examination, a 12-lead electrocardiogram, standard laboratory tests (kidney function, liver function, HbA1c, full blood count), and biomarker sample collection. Randomization and administration of the first trial drug dose occurred within 24 h of the baseline CMR study.

The trial employed a quadruple-blind design to ensure that participants, care providers, outcome assessors, and data analysts were unaware of the treatment allocation. Emergency unblinding was permitted only in situations where knowledge of the participant’s allocation group was essential for managing a serious adverse event or a critical medical situation. The unblinding procedure required the treating physician to contact the trial pharmacist responsible for managing the randomization codes. The randomization code was disclosed solely for the specific participant, and the reason for unblinding was thoroughly documented and reported to the DSMB. Measures were taken to ensure that the study team remained blinded to the allocation, except the treating physician directly involved in the emergency management.

#### 2.5.2. Data Collection Plan

Study data were collected and managed using REDCap electronic data capture tools hosted at Hospital Clínic of Barcelona (redcap.clinic.cat). REDCap (Research Electronic Data Capture) is a secure, web-based software platform designed to support data capture for research studies, providing (1) an intuitive interface for validated data capture; (2) audit trails for tracking data manipulation and export procedures; (3) automated export procedures for seamless data downloads to common statistical packages; and (4) procedures for data integration and interoperability with external sources.

An anonymized and predefined electronic Case Report Form, developed by the investigators, was completed by each participating center. The selected variables focused on baseline characteristics, CMR parameters, biomarkers, medications, and outcomes (Table S1). The principal investigator and the trial statistician have access to the final dataset.

#### 2.5.3. Follow-Up Visits

After randomization, trial visits were scheduled at 1, 3, and 6 months, as outlined in Table 1. All visits were conducted in person. Participants were contacted regularly through scheduled follow-up visits, with reminders sent via telephone prior to each visit. If in-person visits were infeasible, telephone consultations and delayed laboratory sample collection were arranged. Medication adherence was assessed by counting pills at each follow-up visit. Outcome data, including CMR, were also collected for participants who discontinued or deviated from the protocol, when feasible.

All participants received standard post-MI care in accordance with international guidelines, including beta-blockers, angiotensin-converting enzyme inhibitors or angiotensin receptor blockers, statins, and dual antiplatelet therapy [10]. Participation in routine cardiac rehabilitation programs was encouraged. The use of diuretics and glucose-lowering agents was permitted when clinically indicated. Initiation of SGLT2is outside the trial protocol was prohibited. After the study ended, subsequent treatment was determined at the discretion of the treating physician for each participant.

#### 2.5.4. Cardiac Magnetic Resonance Imaging

CMR was performed at baseline and 6 months following randomization on a 3T scanner (ARCHITECT, General Electric, Milwaukee, Wisconsin, 53223, USA) equipped with a phased-array body surface coil and a cardiac-dedicated package with electrocardiogram gating. The following images were obtained within the study: conventional steady-state free-precession cine images covering the entire left ventricle on the short axis, with a slice thickness of 8 mm and no gap between slices, to calculate the indexed end-diastolic volume, indexed end-systolic volume, LVEF, and LV mass. T2 maps were obtained prior to contrast injection on the short axis. T1 maps were acquired before gadobutrol administration (0.2 mmol/kg, Gadovist^®^, Bayer Hispania, Barcelona, 08970, Spain) and 15 min later using a modified Look-Locker inversion-recovery sequence (MOLLI) with an embedded motion correction algorithm in the short-axis orientation at the basal, mid-ventricular, and apical levels, as well as in the four- and two-chamber views. Pre-contrast T1 maps were obtained using a 5-(3)-3 scheme, and post-contrast T1 maps were obtained with a 4(1)3(1)2 scheme. Late enhancement images were acquired 10 min after gadolinium administration using a standard ECG-gated T1-weighted segmented inversion-recovery gradient-echo sequence during repeated breath-holds in the same short axis locations and long axis views as for the cine images.

All scans were analyzed at a core laboratory facility (IDIBAPS Cardiac Imaging Group, Barcelona, 08006, Spain) by experienced cardiologists (CIM and JTO) in CMR postprocessing to generate a clinical report of the study. The scans were then pseudo-anonymized and analyzed by a single operator in accordance with the guidelines from the Society for Cardiovascular Magnetic Resonance and the European Society of Cardiovascular Imaging for reporting CMR examinations [11]. The baseline and 24-week scans were analyzed in pairs and side-by-side to minimize intra-observer variability, with the operator blinded to treatment allocation.

##### Native T1 and ECV Value Derivation

Whenever possible, three regions of interest (ROIs) of at least 1 cm^2^ were drawn in three distinct LV myocardial segments in the remote non-infarcted myocardium among the basal and midventricular slices, avoiding areas with image artifacts. Special attention was given to appropriate co-registration of the tracings between the baseline and follow-up studies to minimize interstudy variability and misregistration. The corresponding late-gadolinium enhancement short-axis images in the T1 maps were checked to avoid placing ROIs in areas with acute or chronic myocardial infarction. The mean values of all ROIs were labeled as the remote native T1 values for the baseline and follow-up studies. The mean myocardial ECVs for both baseline and follow-up studies were computed using the following formula:

(1) $$ECV=\left(1-hematocrit\right)\times \left\lceil \frac{1}{Postcontrast\;T1my}-\frac{1}{Native\;T1my}\right\rceil ÷\left\lceil \frac{1}{Postcontrast\;T1bp}-\frac{1}{Native\;T1bp}\right\rceil$$

where postcontrast T1my is postcontrast myocardial T1; nativeT1my is native myocardial T1; postcontrast T1bp is postcontrast blood pool T1; and native T1bp is native blood pool T1.

LV Function, Volumes, and Infarct Size Calculations

Circle Cardiovascular Imaging 42 Medical Image post-processing software for Cardiac Magnetic Resonance, Version 5.12.1 (Calgary, Alberta, Canada), was used for these calculations. Briefly, an artificial intelligence-based algorithm automatically delineated the endocardial and epicardial contours at end-systole and end-diastole. Manual corrections were performed, if necessary, to compute the LV end-systolic and end-diastolic volumes and LVEF.

The infarct zone was quantified semi-automatically. Initially, the region with the highest signal intensity in the infarct core was identified, and the infarcted tissue was defined as the area with signal intensity above 50% of the maximum pixel intensity (full width at half maximum intensity method). Regions of microvascular obstruction were included in the quantification. Finally, the infarcted areas were summed and divided by the total mass of the left ventricular wall to calculate the infarct size as a percentage of the total LV mass.

#### 2.5.5. Biomarkers

Venous blood and urine samples for biomarker analysis were collected at 3 timepoints: baseline (i.e., after consent and prior to randomization) and at three and six months following randomization. Blood samples were collected in serum separation BD Vacutainer^®^ SST™ (Becton, Dickinson and Company, Franklin Lakes, NJ, USA) Tubes, immediately chilled, and then centrifuged as soon as possible at 2500× *g* and 4 °C for 10 min. The samples were aliquoted and stored at −80 °C at the laboratory of Experimental Cardiology (IDIBAPS, Barcelona, Spain) until batch analysis. Upon completion of the trial, the samples were analyzed to determine the concentration of C-terminal propeptide of type I procollagen, N-terminal propeptide of type III procollagen, Galectin-3, and Suppression of Tumorigenicity 2. The levels of C-terminal propeptide of type I procollagen and N-terminal propeptide of type III procollagen were determined by an enzyme-linked immunoassay (ELISA) kit (Elabscience Biotechnology Co., Ltd., Houston, TX, USA). Galectin-3 and Suppression of Tumorigenicity 2 were determined by a bead-based multiplex immunoassay (R&D Systems, Inc., Minneapolis, MN, USA).

#### 2.5.6. Drug Discontinuation During the Trial

Participants who initiated an open-label SGLT2i or who were withdrawn from the trial were asked to complete the follow-up CMR.

### 2.6. Trial Outcomes

**Figure 1:**
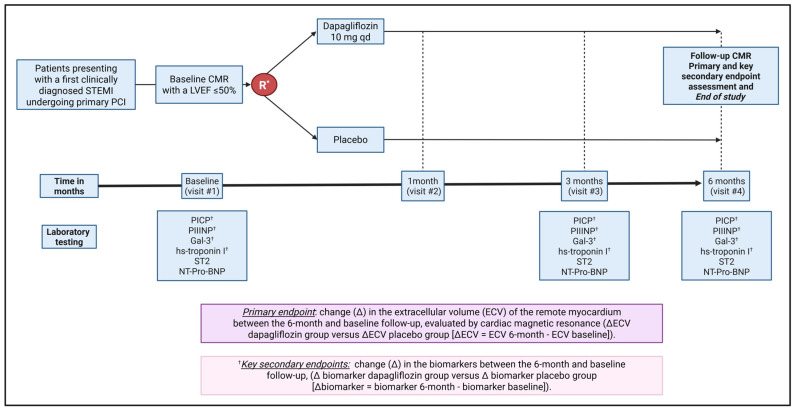
Study design of the DAPA-STEMI trial. * Randomization is stratified by MI localization (anterior vs. non-anterior) and the presence of T2DM (yes vs. no). † Indicates key secondary endpoints. Abbreviations: CMR, cardiac magnetic resonance; ECV, extracellular volume; Gal-3, galectin-3; hs-troponin I, high-sensitivity troponin I; LVEF, left ventricular ejection fraction; MI, myocardial infarction; NT-proBNP, N-terminal pro-B-type natriuretic peptide; PCI, percutaneous coronary intervention; PICP, C-terminal propeptide of type I procollagen; PIIINP, N-terminal propeptide of type III procollagen; qd, once daily; R, randomization; STEMI, ST-segment elevation myocardial infarction; ST2, suppression of tumorigenicity 2; T2DM, type 2 diabetes mellitus.

The primary, secondary, and exploratory outcomes will be measured as the change from baseline to 6 months of follow-up (Figure 1). Differences between the dapagliflozin and placebo groups in these changes will be analyzed. All CMR measurements will be indexed to body surface area, calculated at the time of the scan using the Mosteller formula.

*Primary outcome*

The primary endpoint is the change in ECV of the remote myocardium from baseline to six months, as measured by CMR.

*Secondary outcomes*

These include changes in the circulating biomarkers from baseline to six months:

- C-terminal propeptide of type I procollagen;
- N-terminal propeptide of type III procollagen;
- Galectin-3;
- High-sensitivity troponin I.

*Exploratory outcomes*

The exploratory outcomes include changes in other CMR measures of cardiac function and remodeling from baseline to six months, including:

- Left ventricular end-diastolic and end-systolic volumes;
- Left ventricular mass;
- Left ventricular ejection fraction.

They also include changes in other circulating biomarkers from baseline to six months and clinical events:

- N-terminal pro-B-type natriuretic peptide.
- Suppression of Tumorigenicity 2.
- Rate of major adverse cardiovascular events (MACE), including cardiovascular death, non-fatal myocardial infarction, non-fatal stroke, and heart failure hospitalization. All these endpoints will be adjudicated independently by a clinical event committee (CEC) (Supplementary Materials).
- Adverse events (AEs) and serious AEs were actively collected at follow-up visits and through spontaneous reporting by participants during the study. AEs of interest were represented by severe hypoglycemia, acute kidney injury, acute liver function profile impairment, severe dehydration, symptomatic and sustained hypotension, complicated genitourinary infections, diabetic ketoacidosis, and diabetic foot complications (in diabetic patients). AEs were recorded using a standardized form and assessed for severity, causality, and expectedness. All adverse events were communicated to the DSMB.
- Change in body weight from baseline to six months.

## 3. Statistical Considerations

Based on previous studies conducted by this research group [2,4], it was determined that the change in the ECV of the remote myocardium, measured by cardiac magnetic resonance, between baseline and six months of follow-up in patients with STEMI was 1.5%, with a standard deviation of 2.2%. Assuming a change of 1.5% ± 2.2 in the ECV for the placebo group and an 85% relative reduction in the experimental group, the inclusion of 94 participants (47 per group) would have provided an 80% power to detect such a difference, with an alpha error of 0.05. To account for a 20% loss to follow-up and to ensure balanced groups for stratification based on two variables with two categories each, the final sample size was set at 120 participants (60 per treatment group). After premature termination of the study, a statistical power recalculation was conducted with the same assumption, finding that the final sample of 54 patients would provide 55% power to detect such a difference, with an alpha error of 0.05.

Analyses will be conducted on an intention-to-treat basis. The primary analysis will compare the mean change in ECV of the remote myocardium from baseline to six months between treatment groups (ΔECV dapagliflozin vs. ΔECV placebo) using Student’s t-test or a Mann–Whitney U test, depending on whether the variable has a normal distribution. In addition, a sensitivity analysis will be performed with a mixed model for repeated measures. Similar methods will be used for the analysis of secondary and exploratory outcomes, including changes in circulating biomarkers and other CMR-derived parameters. The mean differences will be reported along with 95% confidence intervals and two-sided *p*-values. Missing data will be handled by the last-observation-carried-forward methodology, while baseline missing data will be considered to be permanently missing. A detailed statistical analysis plan will be finalized prior to database locking and unblinding of the randomized groups.

### 3.1. Monitoring

Independent study monitors (Adelphi, Barcelona, Spain) verified the accuracy and completeness of baseline characteristics, follow-up data, CMR imaging, biomarker results, and event data through audits conducted for all enrolled participants (Supplementary Materials).

### 3.2. Dissemination Plans

All study findings will be published in peer-reviewed scientific journals. Authorship will adhere to the Vancouver guidelines to ensure proper recognition of all contributors. Participants will be informed of the trial results through a lay summary written in clear and accessible Spanish. The study protocol, statistical analysis plan, and results will also be made publicly available. For further details regarding publication, please contact the corresponding author.

## 4. Discussion

The DAPA-STEMI trial was designed to evaluate the potential of dapagliflozin in reducing myocardial fibrosis and improving ventricular function in patients presenting with their first clinically diagnosed STEMI undergoing primary PCI (Graphical Abstract). The study aimed to assess changes in myocardial ECV and circulating biomarkers related to fibrosis over a 6-month follow-up period. A total of 120 participants were initially planned for enrollment. However, due to slow recruitment, the study was halted early, with only 54 participants enrolled.

### 4.1. Anti-Myocardial Fibrosis and Post-MI Effects of SGLT2i

Myocardial fibrosis represents the result of a complex interaction between intra- and inter-cellular signaling networks, resulting in the deposition of extracellular matrix, with transforming growth factor-beta being at the center of these complex interactions [12]. Preclinical studies in heart failure patients have highlighted the presence of a subpopulation of activated fibroblasts in failing hearts, characterized by high expression of extracellular matrix proteins, including type I and III collagen proteins [13], and galectin-3 [14]. Importantly, the availability of specific laboratory kits to detect circulating levels of these biomarkers, as well as the evaluation of the concentration of circulating suppression of tumorigenicity 2 and N-terminal pro-B-type natriuretic peptide [15], has improved the clinical stratification of heart failure patients, as they have been clinically linked to the development and progression of heart failure [12,15].

SGLT2is have demonstrated anti-ischemic, anti-fibrotic, and anti-remodeling effects by reducing extracellular matrix turnover markers and attenuating myocardial fibrosis formation [4,16]. Experimental studies suggest that SGLT2is may prevent ischemia–reperfusion injury through activation of the adenosine 5′-monophosphate-activated protein kinase pathway and reduced production of mitochondrial superoxide production [16], as well as reverse adverse ventricular remodeling by decreasing fibroblast activation and collagen synthesis, likely through altered macrophage and fibroblast gene expression [4,16,17]. Clinical data in patients with T2DM and CAD undergoing CMR indicate reductions in ECV, a surrogate for myocardial fibrosis, and LV mass index with SGLT2is [5]. In post-MI patients, SGLT2is could provide cardiovascular benefits through mechanisms observed in patients with reduced LVEF, including reduced adverse remodeling, preserved ventricular function, and enhanced myocardial energetics via ketone body utilization [18,19,20,21]. Their anti-inflammatory and antioxidant properties mitigate inflammation and oxidative stress, while improved hemodynamics through natriuresis and osmotic diuresis alleviate cardiac workload [22,23]. Additionally, SGLT2is influence ion transport, such as the sodium–hydrogen exchanger, improving calcium handling and contractility [24].

### 4.2. Clinical Evidence for SGLT2is in Post-MI Patients

The benefits of SGLT2is in post-MI patients have been tested in large-scale RCTs, given their high risk for heart failure and adverse remodeling [25]. The Dapagliflozin Effects on Cardiometabolic Outcomes in Patients With an Acute Heart Attack (DAPA-MI) trial evaluated dapagliflozin in 4017 participants with acute MI and without diabetes or prior heart failure [7]. At 1 year, dapagliflozin significantly improved cardiometabolic outcomes compared to the placebo but had no significant impact on cardiovascular death or heart failure hospitalization. Similarly, the Study to Test Whether Empagliflozin Can Lower the Risk of Heart Failure and Death in People Who Had a Heart Attack (EMPACT-MI) trial assessed the role of empagliflozin in 6255 patients with acute MI [26]. At 18 months, empagliflozin did not reduce the risk of the composite outcome of first hospitalization for heart failure or death from any cause compared to placebo. Evidence supporting the use of SGLT2is post-MI remains limited, particularly regarding their effects on STEMI patients and mechanisms such as myocardial fibrosis and ventricular function modification assessed by CMR.

### 4.3. Key Aspects of the Design of the DAPA-STEMI Trial

The DAPA-STEMI trial was designed to address gaps in understanding the effects of SGLT2is on myocardial fibrosis. Prior translational and clinical data suggest that SGLT2is significantly inhibit myocardial fibroblast migration, profibrotic pathways, and extracellular matrix deposition [4,5]. However, these findings are primarily derived from patients with T2DM or heart failure with reduced ejection fraction. The trial focused on myocardial fibrosis by measuring ECV through CMR imaging, an endpoint strongly associated with myocardial fibrosis, cardiac remodeling, and outcomes, including mortality [27]. It enrolled patients presenting with their first clinically diagnosed STEMI undergoing primary PCI with an LVEF ≤ 50%, targeting a population at high risk for progressive ventricular dysfunction, heart failure, and adverse outcomes [25]. The inclusion of patients with and without diabetes ensures broader applicability to real-world practice. The secondary endpoints included circulating fibrosis biomarkers, enabling a detailed evaluation of SGLT2is’ antifibrotic effects. The methodological strengths of this trial include quadruple masking (participant, care provider, investigator, outcomes assessor), core lab imaging, centralized biomarker analysis, and oversight by an independent CEC and DSMB, ensuring rigorous data quality and reliability.

### 4.4. Protocol Amendments and Early Termination

The DAPA-STEMI trial faced significant recruitment challenges, including the COVID-19 pandemic and the rapid adoption of SGLT2is in guideline recommendations. After 37 months, the steering committee decided to terminate the study early due to slow recruitment. A total of 54 patients were finally enrolled, leading to potential lack of statistical power to detect effects on the primary endpoint, as the power recalculation highlighted a 55% power to detect significant differences between groups. Data collection is still ongoing, and analysis has not yet been performed.

Initially, the protocol required an LVEF ≤ 40% to target patients with significant systolic dysfunction, similar to the DAPA-HF trial. However, this was later adjusted to LVEF ≤ 50% to facilitate recruitment, aligning with changes in DAPA-MI and EMPACT-MI [7,26]. Despite these limitations, DAPA-STEMI offers valuable insights into the mechanism of benefits in cardiometabolic outcomes after MI. The trial highlights the ongoing need for targeted treatments to prevent myocardial fibrosis, ventricular remodeling, and heart failure in STEMI patients undergoing primary PCI [25].

## 5. Conclusions

The DAPA-STEMI trial aims to provide critical evidence on whether dapagliflozin can reduce myocardial fibrosis and improve ventricular function in STEMI patients undergoing primary PCI, potentially offering a mechanism for improving cardiometabolic outcomes after MI.

## Figures and Tables

**Table 1:** DAPA-STEMI trial schedule of enrollment, interventions, and assessments.

	Study Period				
	Enrollment	Allocation	Post-Allocation		Close-Out
Timepoint	0–15 Days after Index Myocardial Infarction	0	1 Month	3 Months	6 Months
**Enrollment**:					
Eligibility screen	X				
Informed consent	X				
Baseline cardiac magnetic resonance		X			
Allocation		X			
**Interventions**:					
Dapagliflozin (10 mg)		Start administration	Daily administration	Daily administration	Daily administration
Placebo		Start administration	Daily administration	Daily administration	Daily administration
**Assessments**:					
Physical exam		X	X	X	X
Follow-up cardiac magnetic resonance		X			X
Circulating biomarkers		X		X	X
Adverse events			X	X	X
Pill count			X	X	X

The X represent the time at which each procedure is performed.

## Data Availability

Authorized representatives from the sponsor, host institution, and regulatory authorities will have direct access to trial-related records for monitoring, auditing, and inspection purposes.

## Supplementary Materials

The following supporting information can be downloaded at: https://www.mdpi.com/article/10.3390/jcdd12060220/s1, DAPA-STEMI trial organization details; World Health Organization Trial Registration Details; SPIRIT Checklist for Trials; Table S1. DAPA-STEMI trial collected variables [28].

## Abbreviations

CAD	Coronary Artery Disease
CMR	Cardiac Magnetic Resonance
DAPA-HF	Study to Evaluate the Effect of Dapagliflozin on the Incidence of Worsening Heart Failure or Cardiovascular Death in Patients With Chronic Heart Failure
DAPA-MI	Dapagliflozin Effects on Cardiometabolic Outcomes in Patients With an Acute Heart Attack
DAPA-STEMI	effect of DAPAgliflozin on myocardial fibrosis and ventricular function in patients with STEMI
DSMB	Data Safety Monitoring Board
ECV	Extracellular Volume
GAL3	Galectin-3
HbA1c	Glycated Hemoglobin
IDIBAPS	Institut d’Investigacions Biomèdiques August Pi i Sunyer
LVEF	Left Ventricular Ejection Fraction
MACE	Major Adverse Cardiovascular Events
MI	Myocardial Infarction
PCI	Percutaneous Coronary Intervention
RCT	Randomized Controlled Trial
REDCap	Research Electronic Data Capture
SGLT2i	Sodium–Glucose Cotransporter 2 Inhibitors
STEMI	ST-Segment Elevation Myocardial Infarction
T2DM	Type 2 Diabetes Mellitus
