# Supplementary material for: Effect of DAPAgliflozin on Myocardial Fibrosis and Ventricular Function in Patients with ST-Segment Elevation Myocardial Infarction—DAPA-STEMI Trial
Source: J Cardiovasc Dev Dis. 2025 Jun 11;12(6):220. doi: 10.3390/jcdd12060220 (PMC12194218; doi:10.3390/jcdd12060220)
Supplement: Supplementary file 1 [file jcdd-12-00220-s001.zip › jcdd-3644812-supplementary.pdf]

Supplementary Appendix

Effect of Dapagliflozin in Myocardial Fibrosis and Ventricular Function in Patients with a ST-Segment Elevation Myocardial Infarction, DAPA-STEMI Trial: A structured summary of a study protocol

Ortega-Paz et al.

TABLE OF CONTENTS

DAPA-STEMI trial organization. 2
World Health Organization Trial Registration Details 3
SPIRIT Checklist for Trials 4
Table S1. DAPA-STEMI trial collected variables. 11
References 13

DAPA-STEMI trial organization.

Committee	List of members
**Steering Committee**	Dr. Luis Ortega-Paz (Principal investigator)<br>Dr. Alessandro Sionis (site principal investigator)<br>Dr. Salvatore Brugaletta (Study chair)
**Data Safety Monitoring Board**	Dr. Andrea Ruperti<br>Dr. Riccardo Rinaldi
**Clinical Event Committee**	Dr. Manel Sabaté (Chair)<br>Dr. Víctor Arévalos<br>Dr. Pablo Vidal
**CRO**	Adelphy, Barcelona, Spain
**Monitoring**	Cristina Calle<br>Montserrat Perez
**Statistics**	Effice, Madrid, Spain<br>José Montes
**Core laboratory**	
**Cardiac magnetic resonance**	IDIBAPS Cardiac imaging group, Barcelona, Spain<br>Dr. José Tomás Ortiz-Pérez<br>Dr. Carlos Igor Morr
**Biomarkers**	IDIBAPS, Barcelona, Spain<br>Dr. Ana Paula Dantas

World Health Organization Trial Registration Details

- Trial Registration Number: NCT06619600
- Primary Registry and Trial Identifying Number: EudraCT, 2018-003105-25; ClinicalTrials.gov, NCT06619600
- Date of Registration in Primary Registry: September 14, 2018
- Secondary Identifying Numbers: Institutional Review Board protocol number HCB/2018/0960
- Source(s) of Monetary or Material Support: AstraZeneca (unrestricted grant ESR-19-14489)
- Primary Sponsor: Spanish Society of Cardiology, Ntra. Sra de Guadalupe, 5-7 28028 Madrid, Spain; Tel +34 91 724 23 70
- Contact for Public Queries: Luis Ortega-Paz, Email: Luis.Ortega@jax.ufl.edu
- Contact for Scientific Queries: Luis Ortega-Paz, MD, PhD, Division of Cardiology, University of Florida College of Medicine, Jacksonville, FL, USA; Tel: +1-904-244-3378
- Public Title: Effect of Dapagliflozin in Myocardial Fibrosis and Ventricular Function in STEMI Patients
- Scientific Title: Effect of Dapagliflozin on Myocardial Fibrosis and Ventricular Function in Patients with a ST-Segment Elevation Myocardial Infarction, DAPA-STEMI Trial
- Countries of Recruitment: Spain
- Health Condition(s) or Problem(s) Studied: Myocardial fibrosis and ventricular dysfunction in STEMI patients
- Intervention(s): Dapagliflozin 10 mg daily versus placebo for six months
- Key Inclusion Criteria: Patients aged 30-85 years with STEMI undergoing primary PCI, LVEF ≤50%, hemodynamically stable (Killip Class I), and no contraindication to CMR
- Key Exclusion Criteria: Pregnant or lactating women, type 1 diabetes, prior SGLT2i use, severe liver or kidney disease, malignancy, or contraindications to CMR
- Study Type: Phase III, multicenter, randomized, double-blind, placebo-controlled trial
- Date of First Enrollment: May 27, 2021
- Target Sample Size: 94 patients (53 randomized due to early termination)
- Recruitment Status: Recruitment closed; trial completed
- Primary Outcome(s): Change in extracellular volume (ECV) of the remote myocardium from baseline to six months, measured by CMR
- Key Secondary Outcomes: Changes in circulating biomarkers (e.g., PICP, PIIINP, Galectin-3) and additional CMR parameters (e.g., LV mass, EF)

SPIRIT Checklist for Trials*[28]*

		Reporting Item	Page and Line Number	Reason if not applicable
**Administrative information**				
Title	#1	Descriptive title identifying the study design, population, interventions, and, if applicable, trial acronym	Page 1, Lines 1-3	
Trial registration	#2a	Trial identifier and registry name. If not yet registered, name of intended registry	Page 3, Line 1<br>Appendix page 3, Lines 3-5	
Trial registration: data set	#2b	All items from the World Health Organization Trial Registration Data Set	Appendix page 3, Lines 1-37	
Protocol version	#3	Date and version identifier	Page 6, Lines 15-16	
Funding	#4	Sources and types of financial, material, and other support	Page 6, Lines 10-11<br>Page 23, lines 11-12	
Roles and responsibilities: contributorship	#5a	Names, affiliations, and roles of protocol contributors	Page 1, Lines 4-8<br>Page 23, lines 4-8	
Roles and responsibilities: sponsor contact information	#5b	Name and contact information for the trial sponsor	Appendix page 3, Lines 11-12	
Roles and responsibilities: sponsor and funder	#5c	Role of study sponsor and funders, if any, in study design; collection, management, analysis, and interpretation of data; writing of the report; and the decision to submit the report for publication, including whether they will have ultimate authority over any of these activities	Page 6, Lines 11-12<br>Page 23, Lines 10-12	
Roles and responsibilities: committees	#5d	Composition, roles, and responsibilities of the coordinating centre, steering committee, endpoint adjudication committee, data management team, and other individuals or	Page 6, Lines 6 and 14.<br>Page 16, Line 1<br>Appendix page 2 table	

		groups overseeing the trial, if applicable (see Item 21a for data monitoring committee)		
**Introduction**				
Background and rationale	#6a	Description of research question and justification for undertaking the trial, including summary of relevant studies (published and unpublished) examining benefits and harms for each intervention	Page 5, Lines 1-22	
Background and rationale: choice of comparators	#6b	Explanation for choice of comparators	Page 5, Lines 13-22	
Objectives	#7	Specific objectives or hypotheses	Page 5, Lines 23-24<br>Page 6, Lines 1-2	
Trial design	#8	Description of trial design including type of trial (eg, parallel group, crossover, factorial, single group), allocation ratio, and framework (eg, superiority, equivalence, non-inferiority, exploratory)	Page 5, Lines 23-24<br>Page 6, Lines 1-2	
**Methods: Participants, interventions, and outcomes**				
Study setting	#9	Description of study settings (eg, community clinic, academic hospital) and list of countries where data will be collected. Reference to where list of study sites can be obtained	Page 6, Lines 9-10	
Eligibility criteria	#10	Inclusion and exclusion criteria for participants. If applicable, eligibility criteria for study centres and individuals who will perform the interventions (eg, surgeons, psychotherapists)	Page 7, Lines 18-32<br>Page 8, Lines 1-22	
Interventions: description	#11a	Interventions for each group with sufficient detail to allow replication, including how and when they will be administered	Page 9, Line 13<br>Page 10, Lines 5-12<br>Page 14, Lines 14-16	
Interventions: modifications	#11b	Criteria for discontinuing or modifying allocated interventions for a given trial participant (eg, drug dose change in response to harms, participant request, or improving / worsening disease)	Page 14, Lines 14-16	

Interventions: adherance	#11c	Strategies to improve adherence to intervention protocols, and any procedures for monitoring adherence (eg, drug tablet return; laboratory tests)	Page 11, Lines 6-9.	
Interventions: concomitant care	#11d	Relevant concomitant care and interventions that are permitted or prohibited during the trial	Page 11, Lines 12-18	
Outcomes	#12	Primary, secondary, and other outcomes, including the specific measurement variable (eg, systolic blood pressure), analysis metric (eg, change from baseline, final value, time to event), method of aggregation (eg, median, proportion), and time point for each outcome. Explanation of the clinical relevance of chosen efficacy and harm outcomes is strongly recommended	Page 14, Lines 18-22<br>Page 15, Lines 1-22<br>Page 16, Lines 1-11	
Participant timeline	#13	Time schedule of enrolment, interventions (including any run-ins and washouts), assessments, and visits for participants. A schematic diagram is highly recommended (see Figure)	Page 9, Lines 1-9<br>Page 11, Line 5<br>Table 1	
Sample size	#14	Estimated number of participants needed to achieve study objectives and how it was determined, including clinical and statistical assumptions supporting any sample size calculations	Page 16, Lines 14-22	
Recruitment	#15	Strategies for achieving adequate participant enrolment to reach target sample size	Page 7, Lines 2-15<br>Page 9, Lines 1-9	
**Methods: Assignment of interventions (for controlled trials)**				
Allocation: sequence generation	#16a	Method of generating the allocation sequence (eg, computer-generated random numbers), and list of any factors for stratification. To reduce predictability of a random sequence, details of any planned restriction (eg, blocking) should be provided in a separate document that is unavailable to those who enrol participants or assign interventions	Page 9, Lines 12-22	

Allocation concealment mechanism	#16b	Mechanism of implementing the allocation sequence (eg, central telephone; sequentially numbered, opaque, sealed envelopes), describing any steps to conceal the sequence until interventions are assigned	Page 9, Lines 12-25	
Allocation: implementation	#16c	Who will generate the allocation sequence, who will enrol participants, and who will assign participants to interventions	Page 9, Lines 13-19	
Blinding (masking)	#17a	Who will be blinded after assignment to interventions (eg, trial participants, care providers, outcome assessors, data analysts), and how	Page 10, Lines 4-5	
Blinding (masking): emergency unblinding	#17b	If blinded, circumstances under which unblinding is permissible, and procedure for revealing a participant's allocated intervention during the trial	Page 10, Lines 5-11	
**Methods: Data collection, management, and analysis**				
Data collection plan	#18a	Plans for assessment and collection of outcome, baseline, and other trial data, including any related processes to promote data quality (eg, duplicate measurements, training of assessors) and a description of study instruments (eg, questionnaires, laboratory tests) along with their reliability and validity, if known. Reference to where data collection forms can be found, if not in the protocol	Page 10, Lines 14-23<br>Page 11, Lines 1-23<br>Page 12, Lines 1-23<br>Page 13, Lines 1-23	
Data collection plan: retention	#18b	Plans to promote participant retention and complete follow-up, including list of any outcome data to be collected for participants who discontinue or deviate from intervention protocols	Page 14, Lines 14-16	
Data management	#19	Plans for data entry, coding, security, and storage, including any related processes to promote data quality (eg, double data entry; range checks for data values). Reference to	Page 10, Lines 14-23<br>Page 11, Lines 1-2	

		where details of data management procedures can be found, if not in the protocol		
Statistics: outcomes	#20a	Statistical methods for analysing primary and secondary outcomes. Reference to where other details of the statistical analysis plan can be found, if not in the protocol	Page 17, Lines 1-9	A detailed statistical analysis plan will be finalized prior to database lock and unblinding of the randomized groups.
Statistics: additional analyses	#20b	Methods for any additional analyses (eg, subgroup and adjusted analyses)	Page 17, Lines 1-9	A detailed statistical analysis plan will be finalized prior to database lock and unblinding of the randomized groups.
Statistics: analysis population and missing data	#20c	Definition of analysis population relating to protocol non-adherence (eg, as randomised analysis), and any statistical methods to handle missing data (eg, multiple imputation)	Page 17, Lines 1-9	A detailed statistical analysis plan will be finalized prior to database lock and unblinding of the randomized groups.
**Methods: Monitoring**				
Data monitoring: formal committee	#21a	Composition of data monitoring committee (DMC); summary of its role and reporting structure; statement of whether it is independent from the sponsor and competing interests; and reference to where further details about its charter can be found, if not in the protocol. Alternatively, an explanation of why a DMC is not needed	Page 6, Lines 20-21 Appendix page 2 Table	
Data monitoring: interim analysis	#21b	Description of any interim analyses and stopping guidelines, including who will have access to these interim results and make the final decision to terminate the trial	n/a	No interim analysis was planned. However, if there is a safety concern, the DMC can recommend the steering committee to stop the trial.
Harms	#22	Plans for collecting, assessing, reporting, and managing solicited and spontaneously reported adverse events and other unintended effects of trial interventions or trial conduct	Page 16, Lines 3-10	
Auditing	#23	Frequency and procedures for auditing trial conduct, if any, and whether the process will be independent from investigators and the sponsor	Page 17, Lines 11-14	
**Ethics and dissemination**				

Research ethics approval	#24	Plans for seeking research ethics committee / institutional review board (REC / IRB) approval	Page 6, Lines 5-21	
Protocol amendments	#25	Plans for communicating important protocol modifications (eg, changes to eligibility criteria, outcomes, analyses) to relevant parties (eg, investigators, REC / IRBs, trial participants, trial registries, journals, regulators)	Page 6, Lines 13-15; Page 7, Lines 1-16	
Consent or assent	#26a	Who will obtain informed consent or assent from potential trial participants or authorised surrogates, and how (see Item 32)	Page 7, Lines 5-6	
Consent or assent: ancillary studies	#26b	Additional consent provisions for collection and use of participant data and biological specimens in ancillary studies, if applicable	n/a	There are no ancillary studies.
Confidentiality	#27	How personal information about potential and enrolled participants will be collected, shared, and maintained in order to protect confidentiality before, during, and after the trial	Page 10, Lines 22-23 Page 11, Lines 1-2	
Declaration of interests	#28	Financial and other competing interests for principal investigators for the overall trial and each study site	Page 22, Lines 1-23	
Data access	#29	Statement of who will have access to the final trial dataset, and disclosure of contractual agreements that limit such access for investigators	Page 11, Lines 1-2	
Ancillary and post trial care	#30	Provisions, if any, for ancillary and post-trial care, and for compensation to those who suffer harm from trial participation	Page 11, Lines 17-18	
Dissemination policy: trial results	#31a	Plans for investigators and sponsor to communicate trial results to participants, healthcare professionals, the public, and other relevant groups (eg, via publication, reporting in results databases, or other data sharing arrangements), including any publication restrictions	Page 17, Lines 16-21	

Dissemination policy: authorship	#31b	Authorship eligibility guidelines and any intended use of professional writers	Page 17, Lines 17-18	
Dissemination policy: reproducible research	#31c	Plans, if any, for granting public access to the full protocol, participant-level dataset, and statistical code	Page 17, Lines 19-20<br>Page 23, Lines 15-16	
**Appendices**				
Informed consent materials	#32	Model consent form and other related documentation given to participants and authorised surrogates	n/a	Consent form will be uploaded to clinicaltrials.gov
Biological specimens	#33	Plans for collection, laboratory evaluation, and storage of biological specimens for genetic or molecular analysis in the current trial and for future use in ancillary studies, if applicable	Page 14, Lines 1-12	

Table S1. DAPA-STEMI trial collected variables.

Baseline characteristics	Follow-Up (1-, 3-, 6-months)
Demographics	*Clinical Events*[*]
- Age	- Death (all-cause, cardiovascular)
- Sex	- Recurrent ACS
Comorbidities	- Stroke/TIA
- Hypertension	- Heart failure hospitalization
- Diabetes mellitus	- Bleeding events
- Hypercholesterolemia	- Hypoglycemia/DKA
- Smoking status	- Renal complications
- Chronic kidney disease	- New T2DM diagnosis
- Previous ACS	*Anthropometric Data*[*]
- Previous stroke	- Weight, abdominal circumference
- Heart failure	- Systolic/diastolic BP, heart rate
- History of malignancy	*Medications**
Baseline Medications	- Aspirin, $P2Y_{12}$ inhibitors, beta-blockers
- Aspirin	- Statins, ACE inhibitors/ARBs, ARNI
- $P2Y_{12}$ inhibitors	- Diuretics, MRAs, PPIs
- Beta-blockers	- T2DM treatment (metformin, insulin, etc)
- ACE inhibitors/ARBs	*Laboratories**
- Statins	- Troponin I, glucose, creatinine, HbA1c
Baseline Biomarkers	- Fibrosis biomarkers: PICP, PIIINP, Galectin-3, ST2
- HbA1c, creatinine	*CMR Parameters*[†]
- Troponin I, NT-proBNP	- LVEF
- Fibrosis biomarkers: PICP, PIIINP, Galectin-3, ST2	- LVEDV
CMR Parameters	- LVESV
- LVEF	- Infarct size (%)
- LVEDV	- LV mass

- LVESV	- Microvascular obstruction (mass, %)
- Infarct size (%)	- T1 native global and remote
- LV mass	- Extracellular volume fraction (global, remote)
- Microvascular obstruction (mass, %)	
- T1 native global and remote	
- Extracellular volume fraction (global, remote)	

Data marked with an asterisk (*) will be assessed at all time points (baseline, 1-, 3-, and 6-months). Data marked with a dagger (†) will be assessed at 6 months only. Abbreviations: ACS, acute coronary syndrome; ARBs, angiotensin II receptor blockers; ARNI, angiotensin receptor-neprilysin inhibitor; BP, blood pressure; CMR, cardiac magnetic resonance; DKA, diabetic ketoacidosis; HbA1c, glycated hemoglobin; LVEF, left ventricular ejection fraction; LVEDV, left ventricular end-diastolic volume; LVESV, left ventricular end-systolic volume; MRA, mineralocorticoid receptor antagonist; NT-proBNP, N-terminal pro-B-type natriuretic peptide; PICP, procollagen type I C-terminal propeptide; PIIINP, procollagen type III N-terminal propeptide; PPIs, proton pump inhibitors; ST2, suppression of tumorigenicity 2; T1DM, type 1 diabetes mellitus; T2DM, type 2 diabetes mellitus; TIA, transient ischemic attack.

References

28. Chan AW, Tetzlaff JM, Gotzsche PC, Altman DG, Mann H, Berlin JA, et al. SPIRIT 2013 explanation and elaboration: guidance for protocols of clinical trials. Bmj. 2013;346:e7586. Epub 20130108. doi: 10.1136/bmj.e7586. PubMed PMID: 23303884; PubMed Central PMCID: PMCPMC3541470.
